# Broadly Neutralizing Antibodies for HIV Eradication

**DOI:** 10.1007/s11904-016-0299-7

**Published:** 2016-02-03

**Authors:** Kathryn E. Stephenson, Dan H. Barouch

**Affiliations:** Center for Virology and Vaccine Research, Beth Israel Deaconess Medical Center, Harvard Medical School, E/CLS-1043, 330 Brookline Avenue, Boston, MA 02215 USA; Ragon Institute of MGH, MIT, and Harvard, Boston, MA USA

**Keywords:** HIV, Antibody, Broadly neutralizing antibodies, HIV pathogenesis, Reservoir, bNAb, Eradication, HIV/AIDS, Review

## Abstract

Passive transfer of antibodies has long been considered a potential treatment modality for infectious diseases, including HIV. Early efforts to use antibodies to suppress HIV replication, however, were largely unsuccessful, as the antibodies that were studied neutralized only a relatively narrow spectrum of viral strains and were not very potent. Recent advances have led to the discovery of a large portfolio of human monoclonal antibodies that are broadly neutralizing across many HIV-1 subtypes and are also substantially more potent. These antibodies target multiple different epitopes on the HIV envelope, thus allowing for the development of antibody combinations. In this review, we discuss the application of broadly neutralizing antibodies (bNAbs) for HIV treatment and HIV eradication strategies. We highlight bNAbs that target key epitopes, such as the CD4 binding site and the V2/V3-glycan-dependent sites, and we discuss several bNAbs that are currently in the clinical development pipeline.

## Introduction

More than 78 million people have been infected with HIV, and 39 million people have died since the beginning of the AIDS epidemic [1]. In 2013, there were 1.5 million deaths attributable to HIV infection and 6000 new HIV infections per day. The great majority of new infections (68 %) occurred in sub-Saharan Africa, with large proportions of AIDS-related deaths occurring throughout the world, including in Nigeria (14 %), South Africa (13 %), India (8 %), and the Russian Federation (2 %). One reason that such high rates of AIDS-related deaths continue to occur globally, despite the advent of drugs that are highly effective at suppressing HIV replication, is that only two in five people living with HIV actually have access to antiretroviral therapy (ART). Moreover, ART does not cure HIV infection and must be maintained for a lifetime. Even in the USA, only 30 % of the 1.2 million people living with HIV have suppressed HIV to undetectable levels, despite the fact that most HIV-infected people in the USA have access to ART [2]. As a result, there has been no decline in AIDS-related deaths in the USA for over a decade. Antiretroviral therapy therefore is necessary but not sufficient to end the global AIDS epidemic.

The recent discovery of highly potent, broadly neutralizing, HIV-specific monoclonal antibodies (bNAbs) provides a novel class of potential therapeutic agents. It has long been known that neutralizing antibodies can target the HIV envelope (Env) and effectively suppress viral replication in vitro [3]. Until recently, however, bNAbs were few in number, targeted a narrow spectrum of HIV strains, and were not potent enough for practical use.

Over the last 5 years, the field has changed dramatically: new developments in high throughput single-cell BCR amplification and novel soluble Env selection tools have led to the isolation of new monoclonal antibodies with substantially increased potency and breadth. Phase 1 clinical trials of two CD4 binding site-specific bNAbs, VRC01 and 3BNC117, have shown that these antibodies are well tolerated in HIV-infected and HIV-uninfected adults, and 3BNC117 has been shown to provide antiviral activity in humans [4••, 5••]. Moreover, preclinical data in the non-human primate model using the V3 glycan-dependent bNAb PGT121 demonstrated reduction of proviral DNA in both blood and tissues [6••]. As a result, several laboratories are exploring the possibility that bNAbs may contribute to HIV eradication strategies.

## Early Efforts Utilizing Antibodies to Treat HIV

### Passive Immunotherapy with Pooled Plasma from HIV-Infected Donors

Passive transfer of anti-HIV antibodies has been tested for the treatment of HIV since the late 1980s, when investigators attempted to suppress viral replication with infusions of inactivated hyperimmune plasma pooled from HIV-infected donors [7–12]. Jackson et al. (1988) demonstrated in six subjects that infusions of plasma from donors with high titers of anti-p24 (HIV core antigen) led to clearance of p24 antigen in the blood for up to 11 weeks [9]. Karpas et al. (1988) showed similar results in 10 subjects following infusion of hyperimmune plasma [10, 11]. However, follow-up studies that were randomized and placebo-controlled were less clear in their findings. Jacobson et al. (1993) showed in 65 subjects that monthly infusions of anti-HIV hyperimmune plasma as compared with placebo had no impact on quantitative HIV cultures, CD4 counts, incidence of opportunistic infections, or death [7]. Still, a trend towards longer survival and delayed opportunistic infections was observed. Levy et al. (1994) also showed that in a randomized, placebo-controlled trial of 220 subjects, infusions of hyperimmune plasma led to improved clinical outcomes only in subjects with CD4 counts 50–200 cells/mm^3^ who received the highest dose [13]. In this subset of subjects, a significant increase in CD4 count was observed, but overall, differences in mortality did not reach statistically significance. On the other hand, Vittecoq et al. (1995) observed that passive immunotherapy did lead to a significant delay in the appearance of the first AIDS-defining event in 86 subjects as compared to placebo when given more frequently (every 2 weeks), as well as a mortality benefit [8]. A retrospective analysis of changes in plasma HIV RNA confirmed that after the third dose, there was a significant difference in viral load between the passive immunotherapy and placebo groups [14]. These early studies provided the key observation that infusion of HIV-specific antibodies can lead to reductions in plasma virus that may lead to clinical benefits.

### Passive Immunotherapy with First-Generation Broadly Neutralizing Antibodies

Immunotherapy with anti-HIV antibodies changed in focus in the late 1990s, when the first bNAbs became available for clinical testing. Most clinical trials focused on the three monoclonal antibodies 2G12, 2F5, and 4E10 [15–21]. 2F5 recognizes the ELDKWA motif on the ectodomain of gp41 [22]; 2G12 is directed against N-linked glycans in the C2, C3, V4, and C4 domains of gp120 [23, 24]; and 4E10 binds to a linear epitope (NWFDIT) that is adjacent to the 2F5 binding site on gp41 [25]. Armbruster et al. (2002, 2004) showed in two studies that combinations of these antibodies were safe and well tolerated in 15 HIV-infected subjects at doses of up to 14 g of antibody over a 4-week period [15, 16], and follow-up analyses by Stiegler at al. (2002) demonstrated that combination of these bNAbs led to reductions in plasma HIV RNA of up to 1.46 log10 copies/ml in a subset of volunteers [17].

Based on these results in viremic HIV-infected subjects, Trkola et al. (2005) conducted a proof-of-principle study to evaluate the effect of the three bNAb cocktail (2G12, 2F5, 4E10) in HIV-infected subjects whose virus was well controlled on ART to evaluate their potential impact following cessation of ART [18]. The hypothesis was that the antibodies might work in tandem with host immune responses to delay or to prevent viral rebound. Fourteen HIV-infected participants (8 chronic, 6 acute) on ART received 13 passive infusions of the three bNAb cocktail over 11 weeks, with treatment interruption commencing after the first infusion. Only two of eight chronically treated subjects showed a delay in viral rebound following passive immunization when compared to the kinetics of previous structured treatment interruptions. However, among the acutely treated subjects, there was a significant difference in the time to viral rebound as compared to a historical control group (median 8 vs. 3.75 weeks), and efficacy appeared to correlate with higher plasma concentrations of 2G12. This study also revealed a major limitation of the three bNAb cocktail: 12 of the 14 subjects developed resistance to 2G12 in rebounding virus, highlighting the ease with which viral escape can emerge. This result also suggested that 2G12 was the most (or only) active Ab in the cocktail.

Mehandru et al. (2007) also tested the three bNAb cocktail in HIV-infected subjects on ART (*N* = 10) [26]. Instead of stopping ART after the first infusion, however, ART was continued through three infusions and then stopped. The hypothesis was that the immediate interruption of therapy done by Trkola et al. might not have allowed adequate time for antibodies to reach all tissue compartments of the body. Results were very similar; 8 of 10 subjects demonstrated viral rebound despite 16 weeks of treatment, although the kinetics of rebound was delayed compared to historical data. Again, viral escape with resistance to 2G12 was observed in 7/8 of subjects, particularly with mutations at N-linked glycosylation sites.

## Current Efforts to Develop Antibodies for HIV Eradication

In the last 5 years, there has been an explosion in the number of potent HIV-specific bNAbs, several of which are being explored as therapeutic and prophylactic candidates. Advances in high throughput single-cell B cell receptor amplification, as well as new soluble trimeric Envs that can be used to select bNAbs, have led to the identification of multiple bNAbs with increasing potency and breadth. The bNAbs that are the most advanced in clinical development are outlined below.

### CD4 Binding Site Antibodies

HIV entry into target cells is dependent on viral attachment to the CD4 receptor and is mediated through binding to a conformational epitope on the trimeric Env glycoprotein termed the CD4 binding site (CD4bs). Antibodies that are specific to the CD4bs can therefore block viral entry and inhibit viral replication. Moreover, the CD4bs is functionally conserved across diverse HIV strains, and thus anti-CD4bs antibodies can be extremely broad. Many CD4bs antibodies have now been isolated from human donors, and they share common structural features, such as heavy chain mimicry of the CD4 receptor [27–29].

One of the first CD4bs antibodies identified was VRC01, which was originally isolated from an HIV-infected individual that had been living with untreated infection for over 15 years [30]. Like other CD4bs antibodies, VRC01 is highly somatically mutated, having evolved in response to continual virus escape over many years [31]. Using a panel of 190 Env-pseudotyped viral strains representing all major clades and circulating recombinants, Wu et al. (2010) demonstrated that VRC01 neutralized 91 % of pseudovirions at a half maximal inhibitory concentration (IC50) of <50 μg/ml, and neutralized 72 % of primary isolates at an IC50 of <1 μg/ml [30]. Preclinical challenge studies in non-human primates confirmed that VRC01 had protective activity in vivo as well [32].

Based on these in vitro and preclinical data, the Vaccine Research Center (VRC) at the National Institute of Allergy and Infectious Diseases embarked on a clinical development program of VRC01. One emphasis of the program is to test whether VRC01 might have potential as a prophylactic agent, particularly in the setting of preventing perinatal transmission of HIV [33]. In 2015, Ledgerwood et al. reported the results of the first clinical trial testing VRC01 in humans (VRC602) [4••]. In this study, 28 healthy adults received varying doses of VRC01 ranging from 5 up to 40 mg/kg given as an intravenous infusion, as well as subcutaneously in some subjects at 5 mg/kg. Doses were given either once or twice, 28 days apart, and a subset of subjects received placebo. This study showed that among 43 administrations of VRC01 throughout the study, there were no serious adverse events, and local and systemic solicited reactions were mild. Subjects also did not develop anti-VRC01 antibodies, even though some possessed IgG1 GM allotypes that mismatched with VRC01. The pharmacokinetic analysis showed that VRC01 had a half-life of 15 days when given i.v. with 28-day trough levels that ranged from 35 to 89 μg/ml depending on the dose and frequency of administration. Based on preclinical data that had shown VRC01 was 50 % protective at concentrations of 18–28 μg/ml [32], the authors concluded that potentially protective VRC01 serum levels were observed in their subjects for up to 8 weeks following a second infusion. The potential utility of VRC01 in therapeutic and eradication strategies is currently being explored in HIV-infected subjects on and off antiretroviral treatment, and a phase 2b efficacy study to evaluate VRC01 for prevention of acquisition of HIV is expected to start soon.

3BNC117 is a CD4bs-specific antibody that was isolated from a viremic controller, i.e., an HIV-infected individual with low plasma HIV RNA in the absence of antiretroviral therapy [34]. Scheid et al. (2011) demonstrated that 3BNC117 neutralized 195 out of 237 HIV-1 strains with an average IC50 of 0.08 μg/ml [34]. Shingai et al. (2013) reported that 3BNC117, when given as monotherapy to two non-human primates at a dose of 10 mg/kg, induced a rapid decline of plasma viremia that persisted until 20 days following infusion; results were improved when 3BNC117 was given in combination with the V3 glycan-dependent antibody 10-1074 [35•].

Caskey et al. (2015) then tested the safety and antiviral activity of 3BNC117 when given as an intravenous infusion to 12 HIV-uninfected and 17 HIV-infected adults [5••]. The study was structured as a dose-escalation trial of a single intravenous infusion of 1, 3, 10, or 30 mg/kg of 3BNC117. Caskey et al. demonstrated that 3BNC117, like VRC01, was well-tolerated at all doses. Interestingly, the pharmacokinetic profile of 3BNC117 was different between HIV-infected and HIV-uninfected subjects, with the half-life of the antibody of 9 days in HIV-infected subjects as compared to 17 days in HIV-uninfected subjects. These data suggest that 3BNC117 is cleared more rapidly in the setting of viremia, possibly due to antibodies rapidly binding virions followed by clearance of antigen-antibody complexes. This study also showed that 3BNC117’s antiviral effect was dose dependent. At 1 and 3 mg/kg, 3BNC117 was largely ineffective at reducing plasma HIV RNA, while 30 mg/kg led to a transient 0.8–2.5 log10 drop in plasma HIV RNA. The starting viral load of subjects appeared to influence the magnitude of response and the duration of suppression, and the sensitivity of the circulating virus to 3BNC117 was critical. Analysis for viral escape showed that two of five individuals tested who received the 30 mg/kg dose developed >5-fold reduction of 3BNC117 neutralization sensitivity by 28 days after antibody infusion. The remaining three of five individuals showed little reduction in neutralization sensitivity, suggesting that 3BNC117 resistance can occur quickly but not in all subjects.

### V3 Glycan-Dependent Antibodies

PGT121 is a monoclonal antibody isolated in 2011 from an African donor that targets a V3 glycan-dependent site on the HIV envelope [36]. PGT121 is distinct from other V3-specific monoclonal antibodies, like KD-247, because it does not bind simply to the GPGR region of V3 [37]. Instead, PGT121 has a long heavy chain complementarity determining region (CDR) that forms an antibody binding site with two functional surfaces. Structural studies suggest that PGT121 inhibits CD4 binding to gp120 despite the fact that it does not engage the classically described CD4 binding site [38]. Instead, PGT121 likely interferes with Env receptor engagement by an allosteric mechanism in which key structural elements, such as the V3 base, the N332 oligomannose glycan, and surrounding glycans, including a putative V1/V2 glycan, are locked into a conformation that obstructs CD4 binding [38]. By interfering with a highly conserved function required for viral entry, PGT121 has excellent potency and breadth of activity. For example, Walker et al. (2011) demonstrated that PGT121 had a median IC50 of 0.03 μg/ml when tested against a panel of 162 HIV pseudoviruses; this potency was a log higher than that observed for the CD4-binding site antibody VRC01 (0.32 μg/ml). In addition, Walker et al. demonstrated that PGT121 was able to neutralize 70 % of pseudoviruses tested.

Our laboratory investigated the therapeutic efficacy of PGT121 by giving a single infusion of 10 mg/kg PGT121 alone, 3BNC117 alone, or the control monoclonal antibody DEN3 in rhesus macaques that were chronically infected with SHIV-SF162P3 [6••]. PGT121 alone resulted in rapid virologic control to undetectable levels by day 7, followed by viral rebound by days 42–56 in most animals when serum PGT121 titers dropped to undetectable levels. Rare animals with the lowest starting viral loads exhibited long-term virologic control. PGT121 also appeared to reduce proviral DNA in both blood and tissues [6••]. Moreover, PGT121 resulted in reductions in the percentage of PD-1+Ki67+ Gag-specific CD8+ and CD4+ T lymphocytes, respectively, suggesting that the antibody infusion may have improved the function of host virus-specific T cell responses, likely as a result of the reduction of circulating viral antigens [6••]. This conclusion was supported by the fact that setpoint viral loads following viral rebound were 0.6 log lower than setpoint viral loads prior to mAb infusion, suggesting that mAb infusion may have modulated host immune function [6••].

Viral rebound in the above experiment occurred after a median of 56 days when antibody titers declined to undetectable levels. Rebound virus was sequenced in all animals and no viral escape variants were detected in any animals, suggesting that PGT121 may have a relatively high bar for the development of resistance. Further structural and binding assessments have suggested that PGT121 can make at least four contacts on the Env surface and is not entirely dependent on the N332 glycan. This promiscuous glycan binding results in the need for multiple mutations to escape from this antibody in certain settings [39, 40].

These studies demonstrated that PGT121 exhibited antiviral activity in viremic, SHIV-infected rhesus monkeys, but these data did not directly show targeting of the viral reservoir. To address this question, ongoing studies are evaluating PGT121 in ART-suppressed, SHIV-infected rhesus monkeys. PGT121 is also being advanced into clinical trials, with plans for first-in-human, phase 1 testing in HIV-uninfected and HIV-infected adults in 2016. 10-1074 is a related V3 glycan-dependent antibody that is also undergoing phase 1 testing.

### V2 Glycan-Dependent Antibodies

CAP256 is a recently described bNAb isolated from an HIV-infected individual in South Africa who had been infected (and subsequently superinfected) with HIV-1 subtype C for approximately 5 years before initiating antiretroviral therapy [41]. Moore et al. (2011) demonstrated that CAP256 had a bias towards neutralizing subtype C and A viruses, in some cases with remarkable potency. Epitope mapping studies showed that CAP256 bound a quaternary epitope found on the trimeric form of the envelope glycoprotein that was dependent on residues 159 to 171 in the V2 loop. Analysis of similar antibodies in this class revealed that the long heavy-chain CDR region 3 loops pierce the HIV-1 glycan shield to bind to the quaternary epitope at the apex of the HIV-1 spike where the V1V2 regions of two gp120 protomers meet [42, 43]. Reh et al. (2015) demonstrated that two CAP256 variants (VRC26.08 and VRC26.09) were also able to inhibit cell-cell virus spread [44].

PGDM1400 was recently identified by Sok et al. (2014) by using trimeric HIV-1 envelope (BG505 SOSIP.664) as bait to select memory B cells from an HIV-infected donor by single-cell sorting [45•]. PGDM1400 exhibited exceptional breadth and potency. For example, PGDM1400 neutralized 83 % of viruses of a cross-clade 106-virus panel, with a median IC_50_ of 0.003 μg/ml. Structural studies revealed that PGDM1400, like CAP256, binds to the trimer apex via a very long (34-residue) CDRH3 arm. Moreover, the combination of PGDM1400 + PGT121 neutralized 98 % of tested global viruses at a median IC_50_ of 0.007 μg/ml. These data suggest the potential of bNAb cocktails for global coverage.

## The Future of Broadly Neutralizing Antibodies for HIV Eradication

The current generation of bNAbs against HIV are notable for their potency and breadth, and they have been shown to lead to declines in plasma viremia in preclinical studies and in phase 1 clinical trials [4••, 5••, 6••, 35•]. However, the ability of bNAbs to target the latent viral reservoir and to contribute to viral eradication strategies remains unknown. In vitro data has shown that VRC01 and PGT121 can inhibit viral replication produced from reactivated reservoir cells [46], and mouse studies have shown that FcgR-mediated effector function is required for 3BNC117 to block viral entry and suppress viremia [47]. Moreover, PGT121 infusion in SHIV-infected non-human primates reduced proviral DNA [6••].

Nevertheless, it is likely that bNAbs will need to be used in combination with latency reversing agents, such as histone deacetylase inhibitors (HDACi) or TLR agonists [48, 49]. Halper-Stromberg et al. (2014) tested the concept of combination bNAb/LRA treatment by administering the bNAbs 3BNC117, 10-1074, and PG16 together with vorinostat (HDACi), I-BET151 (BET protein inhibitor), and CTLA4 (a T cell inhibitory pathway blocker) in humanized HIV-infected mice [50]. The authors found that only 10 of 23 mice that had antibody-induced suppression went on to viral rebound following LRA treatment, and of the remaining 57 % of mice that did not rebound, all had undetectable cell-associated viral RNA. Studies in non-human primates are ongoing to evaluate various bNAb/LRA combination treatments in animals with an intact immune system that may contribute to Fc-mediated cell killing. Klein et al. (2014) reported in humanized mice that bNAbs functioned together with autologous antibodies to achieve durable viral suppression [51]. The strategy of combining bNAbs with LRAs will also be investigated in humans in the next several years.

## Conclusions

The concept of using antibodies against HIV to suppress viral replication has been around for many years, but early efforts using hyperimmune plasma from HIV-infected donors and bNAbs with low potency and narrow breadth were largely disappointing. The advent of multiple new bNAbs with substantially greater potency and breadth has led to greatly expanded possibilities. Preclinical studies have shown that certain bNAbs can reduce viral loads and also lead to reductions of proviral DNA, particularly when given with latency reversal agents. Most importantly, early clinical trials have shown that bNAbs are safe, well-tolerated, and have antiviral activity in HIV-infected subjects. Additional clinical trials are planned for the coming years.

## References

[1] UNAIDS. The Gap Report. (2014).

[2] Centers for Disease Control and Prevention. HIV Surveillance Report. (2014).

[3] Burton DR, Poignard P, Stanfield RL, Wilson IA (2012). Broadly neutralizing antibodies present new prospects to counter highly antigenically diverse viruses. Science.

[4.••] Ledgerwood JE (2015). Safety, pharmacokinetics, and neutralization of the broadly neutralizing HIV-1 human monoclonal antibody VRC01 in healthy adults. Clin Exp Immunol.

[5.••] Caskey M (2015). Viraemia suppressed in HIV-1-infected humans by broadly neutralizing antibody 3BNC117. Nature.

[6.••] Barouch DH (2013). Therapeutic efficacy of potent neutralizing HIV-1-specific monoclonal antibodies in SHIV-infected rhesus monkeys. Nature.

[7] Jacobson JM (1993). Passive immunotherapy in the treatment of advanced human immunodeficiency virus infection. J Infect Dis.

[8] Vittecoq D (1995). Passive immunotherapy in AIDS: a double-blind randomized study based on transfusions of plasma rich in anti-human immunodeficiency virus 1 antibodies vs. transfusions of seronegative plasma. Proc Natl Acad Sci U S A.

[9] Jackson GG (1988). Passive immunoneutralization of human immunodeficiency virus in patients with advanced AIDS. Lancet.

[10] Karpas A (1988). Effects of passive immunization in patients with the acquired immunodeficiency syndrome-related complex and acquired immunodeficiency syndrome. Proc Natl Acad Sci U S A.

[11] Karpas A (1990). Polymerase chain reaction evidence for human immunodeficiency virus 1 neutralization by passive immunization in patients with AIDS and AIDS-related complex. Proc Natl Acad Sci U S A.

[12] Vittecoq D (1992). Passive immunotherapy in AIDS: a randomized trial of serial human immunodeficiency virus-positive transfusions of plasma rich in p24 antibodies versus transfusions of seronegative plasma. J Infect Dis.

[13] Levy J, Youvan T, Lee ML (1994). Passive hyperimmune plasma therapy in the treatment of acquired immunodeficiency syndrome: results of a 12-month multicenter double-blind controlled trial. The Passive Hyperimmune Therapy Study Group. Blood.

[14] Morand-Joubert L (1997). Virological and immunological data of AIDS patients treated by passive immunotherapy (transfusions of plasma rich in HIV-1 antibodies). Vox Sang.

[15] Armbruster C (2002). A phase I trial with two human monoclonal antibodies (hMAb 2F5, 2G12) against HIV-1. Aids.

[16] Armbruster C (2004). Passive immunization with the anti-HIV-1 human monoclonal antibody (hMAb) 4E10 and the hMAb combination 4E10/2F5/2G12. J Antimicrob Chemother.

[17] Stiegler G (2002). Antiviral activity of the neutralizing antibodies 2F5 and 2G12 in asymptomatic HIV-1-infected humans: a phase I evaluation. Aids.

[18] Trkola A (2005). Delay of HIV-1 rebound after cessation of antiretroviral therapy through passive transfer of human neutralizing antibodies. Nat Med.

[19] Joos B (2006). Long-term multiple-dose pharmacokinetics of human monoclonal antibodies (MAbs) against human immunodeficiency virus type 1 envelope gp120 (MAb 2G12) and gp41 (MAbs 4E10 and 2F5). Antimicrob Agents Chemother.

[20] Morris GC (2014). MABGEL 1: first phase 1 trial of the anti-HIV-1 monoclonal antibodies 2F5, 4E10 and 2G12 as a vaginal microbicide. PLoS ONE.

[21] Gunthard HF (1994). A phase I/IIA clinical study with a chimeric mouse-human monoclonal antibody to the V3 loop of human immunodeficiency virus type 1 gp120. J Infect Dis.

[22] Purtscher M (1994). A broadly neutralizing human monoclonal antibody against gp41 of human immunodeficiency virus type 1. AIDS Res Hum Retrovir.

[23] Trkola A (1995). Cross-clade neutralization of primary isolates of human immunodeficiency virus type 1 by human monoclonal antibodies and tetrameric CD4-IgG. J Virol.

[24] Trkola A (1996). Human monoclonal antibody 2G12 defines a distinctive neutralization epitope on the gp120 glycoprotein of human immunodeficiency virus type 1. J Virol.

[25] Stiegler G (2001). A potent cross-clade neutralizing human monoclonal antibody against a novel epitope on gp41 of human immunodeficiency virus type 1. AIDS Res Hum Retrovir.

[26] Mehandru S (2007). Adjunctive passive immunotherapy in human immunodeficiency virus type 1-infected individuals treated with antiviral therapy during acute and early infection. J Virol.

[27] Scheid JF (2011). Sequence and structural convergence of broad and potent HIV antibodies that mimic CD4 binding. Science.

[28] Zhou T (2013). Multidonor analysis reveals structural elements, genetic determinants, and maturation pathway for HIV-1 neutralization by VRC01-class antibodies. Immunity.

[29] Wu X (2012). Selection pressure on HIV-1 envelope by broadly neutralizing antibodies to the conserved CD4-binding site. J Virol.

[30] Wu X (2010). Rational design of envelope identifies broadly neutralizing human monoclonal antibodies to HIV-1. Science.

[31] Wu X (2015). Maturation and diversity of the VRC01-antibody lineage over 15 years of chronic HIV-1 infection. Cell.

[32] Pegu A (2014). Neutralizing antibodies to HIV-1 envelope protect more effectively in vivo than those to the CD4 receptor. Sci Transl Med.

[33] Voronin Y (2014). HIV monoclonal antibodies: a new opportunity to further reduce mother-to-child HIV transmission. PLoS Med.

[34] Scheid JF (2011). Sequence and structural convergence of broad and potent HIV antibodies that mimic CD4 binding. Science.

[35.•] Shingai M (2013). Antibody-mediated immunotherapy of macaques chronically infected with SHIV suppresses viraemia. Nature.

[36] Walker LM (2011). Broad neutralization coverage of HIV by multiple highly potent antibodies. Nature.

[37] Matsushita S (2015). Passive transfer of neutralizing mAb KD-247 reduces plasma viral load in patients chronically infected with HIV-1. Aids.

[38] Julien J-P (2013). Broadly neutralizing antibody PGT121 allosterically modulates CD4 binding via recognition of the HIV-1 gp120 V3 base and multiple surrounding glycans. PLoS Pathog.

[39] Sok D (2014). Promiscuous glycan site recognition by antibodies to the high-mannose patch of gp120 broadens neutralization of HIV. Sci Transl Med.

[40] Julien J-P (2013). Crystal structure of a soluble cleaved HIV-1 envelope trimer. Science.

[41] Moore PL (2011). Potent and broad neutralization of HIV-1 subtype C by plasma antibodies targeting a quaternary epitope including residues in the V2 loop. J Virol.

[42] Doria-Rose NA (2014). Developmental pathway for potent V1V2-directed HIV-neutralizing antibodies. Nature.

[43] McLellan JS (2011). Structure of HIV-1 gp120 V1/V2 domain with broadly neutralizing antibody PG9. Nature.

[44] Reh L (2015). Capacity of broadly neutralizing antibodies to inhibit HIV-1 cell-cell transmission is strain- and epitope-dependent. PLoS Pathog.

[45.•] Sok D (2014). Recombinant HIV envelope trimer selects for quaternary-dependent antibodies targeting the trimer apex. Proc Natl Acad Sci U S A.

[46] Chun T-W (2014). Broadly neutralizing antibodies suppress HIV in the persistent viral reservoir. Proc Natl Acad Sci U S A.

[47] Bournazos S (2014). Broadly neutralizing anti-HIV-1 antibodies require Fc effector functions for in vivo activity. Cell.

[48] Barouch DH, Deeks SG (2014). Immunologic strategies for HIV-1 remission and eradication. Science.

[49] Euler Z, Alter G (2015). Exploring the potential of monoclonal antibody therapeutics for HIV-1 eradication. AIDS Res Hum Retrovir.

[50] Halper-Stromberg A (2014). Broadly neutralizing antibodies and viral inducers decrease rebound from HIV-1 latent reservoirs in humanized mice. Cell.

[51] Klein F (2014). Enhanced HIV-1 immunotherapy by commonly arising antibodies that target virus escape variants. J Exp Med.

